# Inequity of care provision and outcome disparity in autoimmune hepatitis in the United Kingdom

**DOI:** 10.1111/apt.14968

**Published:** 2018-09-18

**Authors:** Jessica K. Dyson, Lin Lee Wong, Theophile Bigirumurame, Gideon M. Hirschfield, Stuart Kendrick, Ye H. Oo, Ansgar W Lohse, Michael A. Heneghan, David E. J. Jones, the UK‐AIH Consortium, Shirley English, Graeme Alexander, George Mells, Debabrata Majumdar, Vinay Sathyanarayana, John Ramage, Christopher Shorrock, James Maggs, David Elphick, Chris Macdonald, Matthew Cramp, Joanne Sayer, James Jupp, Jessica Dyson, Coral Hollywood, Alexandra Daley, Lynsey Corless, Darren Craig, Jane Collier, Michael Heneghan, Sharat Misra, Chris Corbett, John Dillon, Simon Rushbrook, Thomas Lee, Nicholas M Sharaer, Kara Rye, Andrew Fowell, Andrea Broad, Dina Mansour, Andy Douds, Stephen Ryder, Richard Keld, Earl Williams, William Stableforth, Andrew Austin, Dermot Gleeson, Kenneth Simpson, Imran Patanwala, Alison Brind, Shanika de Silva, Aqueel Jamil, Saket Singhal, Chin Lye Ch'ng, Joanne Topping, Mark Wright, Talal Valliani, Rebecca Jones, Harriet Mitchison, Douglas Thorburn, Aftab Ala, Ye Htun Oo, Sushma Sakena, Francisco Porras‐Perez, Jane Metcalf, Stephen Mitchell, Esther Unitt, Victoria Gordon, Jeremy Shearman

**Affiliations:** ^1^ NIHR Biomedical Research Centre Newcastle University Newcastle upon Tyne UK; ^2^ Department of Hepatology Freeman Hospital The Newcastle upon Tyne NHS Foundation Trust Newcastle upon Tyne UK; ^3^ Institute of Health and Society Newcastle University Newcastle upon Tyne UK; ^4^ Centre for Liver Research NIHR Biomedical Research Centre University of Birmingham & University Hospital Birmingham NHS Foundation Trust Birmingham UK; ^5^ Research and Development GlaxoSmithKline (GSK) Hertfordshire UK; ^6^ I. Department of Medicine University Medical Center Hamburg‐Eppendorf Hamburg Germany; ^7^ Institute of Liver Studies King's College Hospital London UK

## Abstract

**Background:**

Treatment paradigms in autoimmune hepatitis (AIH) have remained largely unchanged for decades. Studies report ≤20% of patients have sub‐optimal treatment response with most requiring long‐term therapy.

**Aim:**

The United Kingdom Autoimmune Hepatitis (UK‐AIH) study was established to evaluate current treatment practice and outcomes, determine the unmet needs of patients, and develop and implement improved treatment approaches.

**Methods:**

The United Kingdom Autoimmune Hepatitis study is a cross‐sectional cohort study examining secondary care management of prevalent adult patients with a clinical diagnosis of autoimmune hepatitis. Enrolment began in March 2014. Prevalent cases were defined as having been diagnosed and treated for >1 year. Demographic data, biochemistry, treatment history and response, and care location were collected.

**Results:**

In total, 1249 patients were recruited; 635 were cared for in transplant units and 614 in non‐transplant centres (81% female with median age at diagnosis 50 years). Overall, 29 treatment regimens were reported and biochemical remission rate was 59%. Remission rates were significantly higher in transplant compared to non‐transplant centres (62 vs 55%, *P* = 0.028). 55% have ongoing corticosteroid exposure; 9% are receiving prednisolone monotherapy. Those aged ≤20 years at diagnosis were more likely to develop cirrhosis and place of care was associated with an aggressive disease phenotype.

**Conclusions:**

There are significant discrepancies in the care received by patients with autoimmune hepatitis in the UK. A high proportion remains on corticosteroids and there is significant treatment variability. Patients receiving care in transplant centres were more likely to achieve and maintain remission. Overall poor remission rates suggest that there are significant unmet therapeutic needs for patients with autoimmune hepatitis.

## 1 INTRODUCTION

Autoimmune hepatitis (AIH) is a progressive inflammatory condition of the liver that may present in either acute or chronic forms.^1^, ^2^, ^3^ If not effectively treated it can progress rapidly to acute liver failure or the development of cirrhosis. With a prevalence of approximately 17 per hundred thousand in Northern European populations the disease affects both adults and children.^4^, ^5^ Treatment paradigms established in the 1970s and 1980s utilising corticosteroids and azathioprine to achieve and sustain remission have remained largely unchanged over several decades.^1^ Although case series of patients treated in specialist centres suggest that disease remission (even using the current definition of normalised transaminase and immunoglobulin G [IgG] levels) can be achieved in up to 80% of patients,^6^ there is concern that real world disease outcomes in patients treated across the spectrum of health care settings may be substantially worse.^7^ This leaves patients at risk of progression to end‐stage disease for which liver transplantation is the only effective therapy.^8^, ^9^, ^10^

International treatment guidelines have defined initial management and ongoing treatment models in AIH.^8^, ^9^, ^10^ All recommend corticosteroids in the form of prednis(ol)one or budesonide with azathioprine. The goals of treatment for patients are biochemical and histological remission, with effective control of symptoms, followed by long‐term maintenance of the remission state. Ideally, this should be achieved with minimisation of the dose of corticosteroid with full withdrawal being the goal. Maintenance with azathioprine monotherapy where possible is advised in UK and European Guidelines to minimise corticosteroid side effects and their impact on quality of life. The majority of patients require long‐term therapy to prevent relapse^7^, ^11^ and increasing numbers of patients suffer with unpleasant side‐effects, poorly controlled disease and a life‐long immunosuppression burden.^12^, ^13^, ^14^, ^15^

The evidence base for management of patients who are nonresponders to conventional immunosuppression is limited. Alternative immunotherapy, whilst recommended as second‐ and third‐line treatment in patients intolerant of azathioprine, can be variable in efficacy and tolerability.^16^, ^17^, ^18^, ^19^, ^20^ In AIH, the majority of data relating to treatment outcomes is derived from large referral centres.^12^, ^21^ Even amongst these expert centres, significant differences exist in relation to approach to treatment.^22^

In recent years, there has been a growing awareness of inadequacies in service provision for many common liver diseases and attention focused on the public health issues pertaining to the burden of liver disease in the UK.^23^, ^24^, ^25^ To date, however, little attention has been given to rarer liver diseases such as primary biliary cholangitis (PBC), primary sclerosing cholangitis (PSC) and AIH in these documents and the unmet needs and requirements of these patient groups, whilst known, have never been properly quantified.^26^, ^27^

Since the majority of data defining outcome and treatment of AIH are derived from tertiary centres which may not accurately reflect the full spectrum of care delivery we set out to derive a national cohort of patients representing multiple hospital practice settings in the UK. The intention of the United Kingdom Autoimmune Hepatitis (UK‐AIH) consortium is to use this platform to define current “real world” practice in the management of AIH within the UK, and to develop, evaluate and implement improved approaches to treatment. The goals of this study are to evaluate current treatment practice and remission rates and determine the real‐life unmet clinical needs of patients with AIH.

## 2 METHODS

The UK‐AIH platform is a UK‐wide cross‐sectional cohort developed to evaluate the management and outcome of adult patients with AIH in the UK and to facilitate the development, evaluation and implementation of improved therapy. A key aim is to determine the unmet needs of patients with AIH. The UK‐AIH patient cohort is comprised of patients 16 years of age or older who carry a clinical diagnosis of AIH. Patients were enrolled from secondary and tertiary hospital settings from March 2014 to March 2017. The cohort described here was of prevalent patients (ie, diagnosis of AIH was typically several years before enrolment into the cohort).

Patient enrolment into this study was based on what individual clinicians considered an a‐priori diagnosis of AIH. Since patients were prevalent and recruited based on the diagnosis made at clinical presentation (typically several years before study enrolment), no attempt was made to calculate the International Autoimmune Hepatitis Group (IAIHG)^28^ or the simplified IAIHG diagnostic criteria from 2008.^29^

Prevalent cases were identified from clinical records of enrolling hospitals. To be eligible, patients had to have carried a diagnosis of AIH for more than 1 year in their referring hospital. Demographic and clinical data, including risk factors for progressive liver disease, were collected on standardised data collection forms completed by the local managing clinicians. Current biochemical status including alanine aminotransferase (ALT), aspartate aminotransferase (AST), immunoglobulin G (IgG) levels, current therapy, past treatment history related to corticosteroid dose use in the 12 months prior to inclusion in the study, and treatment flares in the last 12 months were collected. Data in relation to diagnostic liver biopsy and disease progression, as defined by progression to cirrhosis during follow‐up, were recorded.

Biochemical remission status at the time of study enrolment (rather than at the time of original diagnosis) was assessed using contemporaneous ALT and IgG values. Upper limits of normal of ALT and IgG were utilised for each recruiting centre. A disease flare was defined as a need to treat an increase in ALT level in the previous 12 months with a higher corticosteroid dose than their maintenance dose or through introduction of corticosteroid therapy in patients on corticosteroid‐free maintenance.

The presence of cirrhosis at the time of diagnosis of disease was determined by findings on liver biopsy coupled with imaging criteria. Development of decompensated liver disease and need for liver transplantation following diagnosis were identified as surrogate markers for disease severity and treatment failure. In addition to the information gathered from clinicians, patients completed questionnaires in relation to the prevalence of additional autoimmune disorders, ethnicity, height and weight. A protocol amendment to the study in November 2015 allowed for additional data collection, comprising variables at diagnosis, including autoantibody titres, IgG levels and viral serology including hepatitis A, B, C, E viruses, Cytomegalovirus, and Epstein‐Barr virus.

The protocol was approved by the National Health Service (NHS) Health Research Authority (IRAS ID: 144806, REC reference: 14/LO/0303) and was conducted in accordance with the International Council for Harmonisation (ICH) Good Clinical Practice (GCP) guidelines and the Declaration of Helsinki. Written informed consent regarding the use of data was obtained. Data were analysed using spss version 22, GraphPad Prism 7 and sas 9.4. Nonparametric data are presented as median and range. Continuous variables were described as median, minimum and maximum. Difference between proportions were analysed using the *Z* test. Logistic regression was performed to assess risk factors for cirrhosis development. All *P* values reported are two‐sided, and *P* < 0.05 was considered statistically significant.

## 3 RESULTS

### 3.1 Demographics and baseline features

One thousand two hundred and forty‐nine patients were enrolled into the study cohort from 44 centres (seven liver transplant centres and 37 nontransplant centres). 635 patients were under the care of transplant units and 614 under nontransplant centres. The majority of patients were female (1006, 81%). Median age at diagnosis of AIH of enrolled patients was 50 years (range 2‐86 years). Seventy‐nine (6%) of the patients were diagnosed below the age of 16 with 65/79 (82%) being cared for in transplant units.

Patients managed in transplant centres were diagnosed at a younger age than those managed in nontransplant centres (median 42 years [range 2‐86 years]) with 116 (18%) of patients diagnosed at 20 years or younger compared with median age 55 years (range 4‐86 years) and 30 (5%) diagnosed at 20 years or younger (*P* < 0.0001). The duration of follow‐up between disease diagnosis and study enrolment was longer in transplant centres with a median of 8 (1‐57) years versus 6 (1‐41) years.

**Figure 1:**
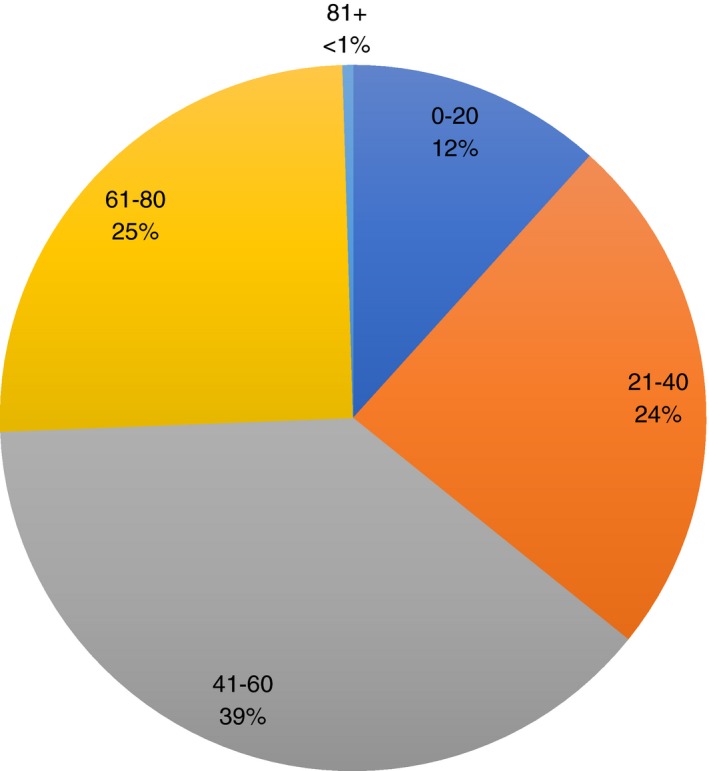
Age at diagnosis in 20 y age brackets for whole cohort (n = 1249), n (%). Patients were categorised according to age at diagnosis; 0‐20 years, 21‐40 y, 41‐60 y, 61‐80 y, 81 y and over

A summary of baseline characteristics (divided into transplant and nontransplant units) at time of study entry is presented in Table 1. In the whole cohort, median body mass index (BMI) at study recruitment was 28.4 kg/m² (range 15.1‐64.0). Figure 1 illustrates the proportion of patients in the study according to age at diagnosis divided into five categories of 20‐year age brackets. In keeping with other studies, the majority of patients were diagnosed between the age of 41 and 60 years.^6^, ^30^, ^31^, ^32^, ^33^

**Table 1:** Baseline characteristics of patients at time of study entry (n = 1249) and patient‐reported presence of other autoimmune conditions (n = 1192, 596 in both types of unit)

	Transplant units	Non‐transplant units
Age at study entry (y), median (range)	52 (17‐91)	63 (18‐95)
Weight (kg), median (range)	75 (40‐169)	76 (38‐165)
Height (cm), median (range)	162.5 (132.5‐192.5)	162.5 (132.5‐192.5)
Body mass index, BMI (kg/m^2^), median (range)	28 (15‐64)	29 (16‐62)

Other autoimmune condition	n (%)	n (%)	*P* value
Thyroid disease	67 (11)	110 (18.5)	0.0004
Rheumatoid arthritis	29 (4.9)	52 (8.7)	0.008
Primary biliary cholangitis	33 (6)	39 (7)	0.47
Ulcerative colitis or Crohn's disease	33 (5.5)	25 (4.2)	0.281
Coeliac disease	17 (2.9)	24 (4)	0.266
Systemic lupus erythematosus	21 (4)	19 (3.2)	0.748
Sjogren's syndrome	15 (2.5)	24 (4)	0.143
Type 1 diabetes mellitus	15 (2.5)	12 (2)	0.559
Primary sclerosing cholangitis	20 (3.4)	7 (1.2)	0.011
Mixed connective tissue disorder	7 (1.2)	4 (0.7)	0.363
Other autoimmune condition	79 (13.3)	100 (16.8)	0.088

Seven hundred and thirty‐four of 742 (99%) of patients with available data were abstinent from alcohol or consumed alcohol within recommended limits in accordance with UK guidelines prior to 2014^34^ (below 15 units per week for women and 22 units per week for men). Of those with available data, 69/739 (9%) were current smokers (less than the 19% of the total adult UK population who are reported to be current smokers by Cancer Research UK) and 221/739 (30%) were previous smokers.

Fifty‐one patients had undergone liver transplantation at time of accession to the study and have been excluded from further analyses (other than for age at diagnosis and the development of cirrhosis) with the denominators reflecting this.

Biochemical data allowing assessment of remission status (defined as normal ALT and IgG at time of study entry) were available for 1114 patients. Of these, 460 (41%) were not in remission at the point of study entry.

### 3.2 Immunosuppression regimens

Table 2 summarises treatment regimens, biochemical remission rates and the number of patients with cirrhosis at diagnosis. Patients were stratified according to the treatment regimen. Overall, 29 different treatment regimens were reported, not including dose variations, transplant patients or those not receiving any treatment for their AIH.

**Table 2:** Summary of treatment regimens, biochemical remission rates (normal ALT and IgG) and number of patients cirrhotic at diagnosis according to treatment regimen (n = 1198, excluding liver transplant patients)^a^

Simplified drug regimen	Number of patients (%)	Number in biochemical remission (%)	Number cirrhotic at diagnosis (%)
Azathioprine/MP alone	392 (33)	252/362 (70)	82/385 (21)
Azathioprine/MP + prednisolone	316 (26)	175/299 (59)	78/309 (25)
Prednisolone alone	103 (9)	43/85 (51)	28/98 (29)
Budesonide alone	19 (2)	5/19 (26)	2/19 (11)
No immunosuppression	85 (7)	51/79 (65)	15/84 (18)
MMF/MA alone	58 (5)	35/54 (65)	11/57 (19)
MMF/MA + prednisolone	112 (9)	53/107 (50)	34/112 (30)
CNI‐containing regimen^b^	56 (5)	16/54 (29)	19/56 (34)
Triple immunosuppression	28 (2)	5/27 (19)	9/27 (33)
Other immunosuppression regimen	53 (4)	24/51 (47)	3/53 (6)

ALT: alanine aminotransferase; IgG: immunoglobulin G; MP: mercaptopurine; MMF: mycophenolate mofetil; MA: myphenolic acid; CNI: calcineurin inhibitor.

a All available data presented but some missing data resulting in denominators for the calculation of biochemical remission and numbers cirrhotic at diagnosis being smaller than the total number of included patients.

b Some patients on a CNI‐containing regimen were receiving triple immunosuppression so appear in both groups resulting in n > 1198.

Despite treatment guidelines recommending that maintenance corticosteroids not be used, 653 of 1198 patients (55%) were taking long‐term corticosteroid therapy as part of their treatment regimen. There were 124/1198 (10%) patients on immunosuppression with corticosteroids only (103 [9%] in the form of prednisolone) and of these, 57/106 (54%) patients were not in biochemical remission. Patients requiring lower doses of corticosteroids or on no corticosteroids at all were more likely to be in biochemical remission. These data are summarised in Table 3.

**Table 3:** Details regarding long‐term corticosteroid dose and remission rates (n = 1198, excludes transplant patients)^a^

Corticosteroid dose	Number of patients (% of cohort)	Number of patients in biochemical remission (%)
≥Prednisolone 10 mg/d (“High dose”)	172 (14.4)	57/162 (35.2)
≥Budesonide 6 mg/d (“High dose”)	30 (2.5)	7/29 (24)
5 mg > Prednisolone <10 mg/d (“Medium dose”)	97 (8.1)	52/91 (57)
Prednisolone ≤5 mg/d^b^ (“Low dose or no prednisolone”)	929 (77.6)	545/861 (63)
≤ Budesonide 3 mg/d^b^ (“Low dose or no prednisolone”)	1168 (97.5)	647/1085 (60)

a All available data presented but some missing data resulting in denominators for the calculation of biochemical remission being smaller than the total number of included patients.

b Including those on no corticosteroids.

There were 658/1198 (55%) patients taking azathioprine ± prednisolone. The median dose of azathioprine was 1.1 mg/kg/d (range 0.2‐2.8). ALT and IgG data were available for 614 patients prescribed azathioprine ± prednisolone, of whom 398 (65%) were in biochemical remission. Biochemical remission rates were lower (256/500 patients, 51% overall) for patients prescribed other treatment regimens. Overall, there were 714 patients taking azathioprine and the dose was available for 696 patients. There were 232 (33%) taking less than the 1 mg/kg/d minimum recommended dose, of whom, 66/220 (30%) patients were not in biochemical remission.

A total of 708 patients (59%) were treated with thiopurines either in the form of azathioprine/mercaptopurine alone (392 patients) or in combination with prednisolone (316 patients, 26% of whole cohort). For patients who were taking thiopurine therapy in isolation, 70% (252/362) were in biochemical remission. In contrast, the rate of biochemical remission for patients receiving thiopurine therapy together with prednisolone was significantly lower at 59% (175/299), *P* = 0.003.

Mycophenolate‐based therapy ± prednisolone (typically used in patients with thiopurine intolerance or nonresponsiveness), were used in 170 patients (14%). 65% (35/54) of patients who received mycophenolate‐based therapy (mycophenolate mofetil [MMF] or myphenolic acid [MA]) in isolation were in biochemical remission. However, MMF or MA were used in conjunction with prednisolone in 112 patients (9% of the total cohort, median dose 1000 mg/d [range 250‐3000]), and of these, only 50% were in biochemical remission, *P* = 0.066).

**Figure 2:**
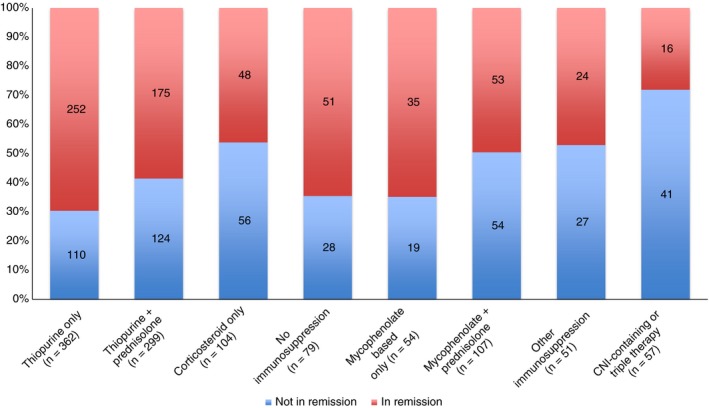
Breakdown of patients by remission status according to simplified drug combinations (excluding transplant patients). CNI: calcineurin inhibitor

Five per cent of the cohort (59 patients) were receiving a calcineurin inhibitor (CNI) or three drug regimen suggesting difficult to control disease. Of these, only 16/57 (28%) were in biochemical remission and 19/58 (39%) had established cirrhosis at diagnosis. Figure 2 summarises complexity of treatment regimen and likelihood of treatment response.

Seven per cent (85) of patients were receiving no immunosuppression and of these, 65% were in remission with 15 (18%) patients being cirrhotic at diagnosis.

### 3.3 Location of care

Of nontransplanted patients (n = 1198), 586 were cared for in transplant units (49%) and 612 in nontransplant units. Treatment regimens utilised in transplant centres were significantly different to those used in nontransplant centres (Table 4). Whilst the total number of combinations of therapy were similar between transplant and nontransplant centres (26 vs 27), patients being cared for in transplant centres were more likely to be on either a triple immunosuppression regimen (*P* = 0.006) including CNI, anti‐CD20 or anti‐Tumour Necrosis Factor (TNF) therapy or a regimen including a CNI (*P* = 0.002).

**Table 4:** Comparisons between patients cared for in transplant units and nontransplant centres and, for nontransplant centres, in centres with and without a specialist hepatologist (n = 1198, transplant patients excluded)

	Transplant unit (n = 586)	Non‐transplant centres (n = 612)	*P* value	Hepatologist (n = 405)	No hepatologist (n = 207)	*P* value
Age of patients at diagnosis, median (range)	42 (2‐86)	55 (4‐86)		55 (2‐86)	55 (7‐79)	
Number of treatment combinations used	26	27		22	18	
Number of patients on regimen including a CNI	39 (6%)	17 (2.9%)	0.002	8 (1.98%)	9 (4.4%)	0.133
Number of patients on triple immunosuppression	21 (3.6%)	7 (1.14%)	0.006	5 (1.2%)	2 (0.97%)	0.759
Not in biochemical remission (ALT and IgG)	214/562 (38%)	246/552 (44.6%)	0.028	156/366 (42.6%)	90/186 (48.4%)	0.199
Maintenance high‐dose corticosteroids during past 12 mo	107/586 (18.3%)	93/612 (15.2%)	0.156	54 (13.3%)	39 (18.8%)	0.085
Attempt to reduce corticosteroid dose during last 12 mo	25/42 (60%)	55/80 (69%)	0.308	35/46 (76.1%)	20/34 (58.8%)	0.101
Previous corticosteroid treatment for disease flare	118/572 (21%)	101/586 (17%)	0.142	67/393 (17%)	34/193 (18%)	0.865
Cirrhotic at diagnosis	145/573 (25.3%)	127/603 (21.1%)	0.085	88/402 (21.9%)	39/201 (19.4%)	0.473

CNI: calcineurin inhibitor; ALT: alanine aminotransferase; IgG: immunoglobulin G.

In nontransplant centres, 405 patients were looked after by a dedicated hepatologist whereas, a gastroenterologist looked after 207 patients. The number of combinations of immunosuppression used by gastroenterologists was fewer than those working as dedicated hepatologists (22 vs 18). In nontransplant units, there was no statistically significant difference between the use of a CNI or triple immunosuppression between hepatologists and gastroenterologists.

A significantly higher proportion of patients treated in transplant centres were in remission compared to nontransplant centres, 62% versus 55% (*P* = 0.028). There was no significant difference in disease flares, the proportion of patients maintained on higher doses of corticosteroids (defined in the study protocol as ≥10 mg of prednisolone per day or budesonide ≥6 mg/d) or the number who had an attempt to reduce their corticosteroid dose in the previous 12 months between transplant and nontransplant units or care under a hepatologist or gastroenterologist.

### 3.4 Development of cirrhosis (includes transplanted patients)

Prevention of the development of cirrhosis in AIH is a major goal of therapy. 289/1223 (24%) of patients were, however, already cirrhotic at the time of diagnosis. Patients diagnosed at 20 years old or younger were significantly more likely to be cirrhotic at diagnosis than patients presenting over the age of 20 (48/141 [34%] vs 241/1082 [22%], *P* = 0.002) although it is unclear whether this represents a more aggressive disease course in children and young adults or a higher likelihood of delayed diagnosis.

Table 5 summarises risk factors for developing cirrhosis. Patients aged 20 years or younger at diagnosis (including transplant patients) were more likely to develop histological or radiological evidence of cirrhosis during follow‐up than patients aged more than 20 years (29% vs 14%, *P* = 0.0007). The total daily azathioprine and corticosteroid dose were not associated statistically with the development of cirrhosis during follow‐up. Whether or not patients were under the care of a hepatologist or gastroenterologist was not associated with disease progression, suggesting that patients with more stable disease were looked after appropriately.

**Table 5:** Risk factors for developing cirrhosis (either histological and/or radiological) during follow‐up from diagnosis to point of study entry (excluding patients who were cirrhotic at diagnosis)

Risk factor		Number developing cirrhosis (%)		Number developing cirrhosis (%)	*P* value
Age at diagnosis^a^	≤20 y	27/94 (29)	>20 y	104/836 (14)	0.0007
Azathioprine dose	<1 mg/kg/d	17/176 (10)	≥1 mg/kg/d	33/344 (10)	0.984
Corticosteroid dose	≥ prednisolone 10 mg and/or budesonide 6 mg/d	18/140 (13)	Prednisolone ≤5 mg and/or budesonide 3 mg/d^b^	80/901 (9)	0.134
Specialist care (for nontransplant unit)	Hepatologist	28/311 (9)	No hepatologist	7/160 (4)	0.070

a Taking the event of liver transplantation as evidence of the development of cirrhosis.

b Includes patients on no corticosteroid.

Table 6 shows the uni‐ and multi‐variate analyses for nontransplanted patients. For these analyses, years since diagnosis has been used instead of age at diagnosis to enable exclusion of the transplanted patients, that is, ensuring that the same patient group is used for each variable and that each patient is only included once in the analyses. Increasing years since diagnosis remained significant as a predictor for development of cirrhosis on uni‐ and multi‐variate analysis, Patients in biochemical remission at study entry were less likely to develop cirrhosis, however, this fell short of statistical significance. When examining treatment regimens (compared to standard therapy with azathioprine ± prednisolone), the use of triple immunosuppression or a CNI‐containing regimen was associated with a higher probability of developing cirrhosis (*P* = 0.011) but this effect became just nonsignificant (*P* = 0.053) on multi‐variate analysis when other risk factors were included in the model. Place of care was associated with a more aggressive disease phenotype with transplant units having a greater proportion of patients developing cirrhosis following their initial diagnosis in both the univariate and multiple logistic regression models.

**Table 6:** Univariate and multiple logistic regression model of risk factors for developing cirrhosis (either histological and/or radiological) during follow‐up from diagnosis to point of study entry (excluding patients who were cirrhotic at diagnosis

Risk factor	Univariate		Multiple	
	Odds ratio	*P* value	Odds ratio	*P* value
Biochemical remission at entry (yes vs no)	0.7033 [0.455, 1.087]	0.1129	0.7514 [0.469, 1.203]	0.2335
Treatment regimen				
Corticosteroid only vs standard^a^	1.8688 [0.955, 3.655]	0.0678	1.5573 [0.76, 3.191]	0.2262
None vs standard	0.9356 [0.383, 2.288]	0.884	0.8025 [0.303, 2.123]	0.6575
Other vs standard	0.9398 [0.535, 1.651]	0.829	0.9743 [0.536, 1.772]	0.9321
Triple or CNI vs standard	2.8558 [1.272, 6.411]	0.011	2.3512 [0.99, 5.586]	0.0528
Transplant unit (yes vs no)	2.3485 [1.484, 3.716]	0.0003	2.0458 [1.258, 3.327]	0.0039
Years since diagnosis	1.1029 [1.075, 1.131]	0.0001	1.0972 [1.069, 1.126]	0.0001

CNI: calcineurin inhibitor.

a Standard therapy = azathioprine ± prednisolone.

## 4 DISCUSSION

In this large, nationwide study of “real world” clinical practice in AIH we have demonstrated both significant limitations in the effectiveness of care for AIH and a high degree of variability in practice and quality between unit types. Our first key observation is that the remission rate using standard criteria is only 59%; a figure falling far short of the benchmark figure of 80% demonstrated to be achievable in specialist centres with a specific interest in disease management and structures in place to deliver optimised care.^6^ Second, over 50% of patients with AIH in the UK are receiving ongoing corticosteroid therapy, in spite of multiple clinical practice guidelines suggesting that maintenance should be with thiopurine monotherapy.^8^, ^9^, ^10^ Third, there was apparent confusion and inconsistency around appropriate treatment with 29 individual treatment regimens described even before allowing for dose variations. Finally, patients receiving care in transplant centres in the UK were more likely to achieve and maintain remission than those looked after in other care locations, despite an apparent bias towards a more severe disease phenotype.

The demographic characteristics and disease associations of the study population were in keeping with previous published series although concurrent PSC was more common in transplant units.^4^, ^32^, ^35^ This suggests that differences in the behaviour of the cohort long term are unlikely to be attributable to the characteristics of the population, but rather, the care received. Risk variation was seen within the cohort with patients presenting at 20 years or younger having the highest rate of cirrhosis at diagnosis and progression to cirrhosis during follow‐up.^36^, ^37^ Disease phenotype profile in this group is in keeping with previously published reports of children with AIH, particularly, Type 2 AIH associated with detectable anti‐Liver Kidney Microsomal (LKM) antibodies in serum.^38^

The rate of corticosteroid use was high in our cohort with 55% of patients remaining on either prednisolone or budesonide. Recently published data from the UK‐AIH study show that the use of corticosteroids is strongly associated with decreased health‐related quality of life that is independent of biochemical remission status.^39^ BMI was high in our cohort, with the median being 28.4 kg/m^2^, and 38% (421/1111) of patients being classified as obese with a BMI > 30 kg/m^2^. This may reflect the overly high use of corticosteroids. Corticosteroid therapy, in conjunction with weight gain, is likely to result in secondary disease development such as the metabolic syndrome, hyperlipidaemia and hypertension in this patient population. What is clear from older data is that when it is possible to entirely withdraw corticosteroids in patients with AIH, an average of 6 kg of weight loss per patient has been reported.^12^

The overall use of thiopurine therapy, either azathioprine or mercaptopurine (±corticosteroid) was 63% (753//1198 patients) of the total patient cohort with 359/753 (48%) taking corticosteroid therapy. This suggests that adherence to established treatment guidelines for AIH patients in the UK (European Association for the Study of the Liver and British Society of Gastroenterology guidelines) is poor. The median dose of azathioprine used in this cohort was only 1.1 mg/kg/d. Thiopurine drug metabolites were not checked in this study (the study protocol precluded this), nor were details of poor treatment tolerance with side effects or adverse events collected. However, we know from published data that an azathioprine dose of up to 2 mg/kg/d can result in enhanced long‐term remission rates in AIH with a concomitant ability to withdraw corticosteroids entirely from the treatment regimen for the majority of patients.^40^

The approach to management of AIH was different between transplant and nontransplant centres. While the overall number of treatment regimens used in transplant compared to nontransplant units was similar (26 vs 27), there was a greater likelihood of patients being exposed to an expanded range of novel treatment options in AIH management (eg, CNIs, three drug regimens or biological agents). This more nuanced and individualised approach to care in the transplant centres appears to translate to less fluctuation in the disease, with more patients in biochemical remission, and consequently is likely to be protective in relation to hepatic outcomes.^6^

This “real world” study of patients with AIH demonstrates and quantifies the therapeutic challenges that have been discussed in the literature.^22^, ^41^, ^42^ The poor remission rates that we report suggest that there are significant unmet needs therapeutically for patients with AIH. For the majority of other autoimmune disorders, significant strides have taken place to facilitate corticosteroid‐free regimens. Examples include the use of disease‐modifying agents and novel antibodies in diseases such as those utilised in Multiple Sclerosis, Inflammatory Bowel Disease and Rheumatoid Arthritis.^43^, ^44^ These conditions are exemplars of being beneficiaries of the novel therapeutics explosion. As an orphan disease with potential hard outcomes such as cirrhosis development, death and liver transplantation, there is a pressing need for novel therapeutic approaches and targets in AIH. Some potential targets for AIH treatment have been explored but none have been realised in clinical practice as yet.^42^ Patients with liver disease, especially those with rare diseases such as AIH, are disadvantaged not just through inertia from the medical community, but are secondarily disadvantaged through an unwillingness of the pharmaceutical industry to offer the use of potentially useful therapeutic agents in clinical trials. As an example, current literature suggests that antibody therapies such as anti‐CD20 or anti‐TNF therapy have only been utilised in <50 patients worldwide with AIH, and even then, only in the context of late disease.^45^, ^46^ In contrast, the alternative treatment paradigm should be one of treating early disease aggressively with potential disease modifiers or stoppers, to avoid life‐long therapy with corticosteroids and other drugs that have been proven outdated for the majority of other inflammatory autoimmune disorders.

Although we believe our data shed important light on the reality of care for AIH in the UK the study has important limitations. The first is that this is an observational cohort study describing outcomes in practice. This limits the scale of the data capture and will clearly miss any very high‐risk patients who died from the disease early in its course and who would thus feature in an incident but not a prevalent cohort. Our approach does avoid, however, the potential for an intensive prospective study to focus attention on, and potentially lead to artificial improvement in, the quality of the care being delivered. The second is that, self‐evidently, the study relates only to practice in the UK. It would of course be of great interest to repeat the approach in other health care settings to explore whether the same limitations in care are present. Anecdote and the limited data available suggest that they are. The third is that our categorisation into transplant units and nontransplant units was a robust but slightly blunt approach. We have tried to account for centres with a specialist interest in AIH (where excellent results are reported^6^) by sub‐dividing nontransplant units into those with and without dedicated hepatologists (data courtesy of Jessica Dyson and Mark Hudson from national survey of liver services). It may be that these centres improve the apparent outcomes in the nontransplant centre group as a whole (as seen with the significantly lower rate of disease flares in centres with a hepatologist), masking the true scale of the care quality divide.

In conclusion, despite its limitations the UK‐AIH cohort demonstrates significant discrepancies in care delivery for patients with AIH. It outlines, in particular, the contrast between real world outcomes for a rare disease cohort and the outcomes achieved in clinical trials. It also suggests that the medical community seems comfortable in accepting both suboptimal patient outcomes and largely outmoded therapeutics for the disorder. This cohort provides evidence of the need to enhance adherence to optimal treatment approaches identified in clinical practice guidelines (through education of both clinicians and patients) and a need for more potent, and patient‐acceptable, therapies for this important condition. Both areas should be research priorities moving forward.

## AUTHORSHIP

*Guarantor of the article*: Jessica Katharine Dyson.

*Author contributions*: LLW, JKD and TB analysed and interpreted the data. JKD, DEJJ, MAH, LLW and TB wrote the first draft of the manuscript. JKD, DEJJ and SK were involved in study concept and design. All authors were involved in the critical revision of the manuscript. All authors approved the final version of the manuscript.
